# The waist circumference-adjusted associations between hyperuricemia and other lifestyle-related diseases

**DOI:** 10.1186/s13098-017-0212-6

**Published:** 2017-02-10

**Authors:** Taiju Miyagami, Hirohide Yokokawa, Kazutoshi Fujibayashi, Toshiaki Gunji, Noriko Sasabe, Mitsue Okumura, Kimiko Iijima, Toshio Naito

**Affiliations:** 1 Department of General Medicine, Juntendo University School of Medicine, Japan Hongo 2-1-1, Bunkyo-ku, Tokyo, 113-8421 Japan; 2 Center for Preventive Medicine, NTT Medical Center Tokyo, Tokyo, Japan

**Keywords:** Hyperuricemia, Metabolic syndrome, Lifestyles, Non communicable disorders, Lifestyle related disorders

## Abstract

**Background:**

Few studies have assessed the associations between hyperuricemia and lifestyle-related diseases after adjusting for waist circumference (WC) and sex.

**Methods:**

This cross-sectional study included 33,498 Japanese individuals, and was conducted at the Center for Preventive Medicine, NTT Kanto Medical Center, Tokyo, from May 2006 to March 2015. Hyperuricemia was defined as a uric acid level of >7 mg/dl in men; >6 mg/dl in women. Metabolic syndrome (Mets) components were defined using the Japanese criteria for Mets. The subjects were stratified into quartiles according to their WC as follows: males: <78.4, 78.4 to <83.5, 83.5 to <89, and ≥89 cm; females: <71.6, 71.6 to <77, 77 to <83.2, and ≥83.2 cm. The relationships between these quartiles and the presence of ≥2 components of Mets or hyperuricemia were then evaluated using Chi square analysis. The presence of ≥2 components of Mets were then determined using multivariate logistic regression analysis adjusting for age, the presence of hyperuricemia, WC, and lifestyle habits.

**Results:**

Hyperuricemia was found to be an independent predictor of lifestyle-related diseases after adjusting for age, WC, and lifestyle in both sexes. Males: a uric acid level of >7 mg/dl (odds ratio [OR]: 1.70, 95% confidence interval [CI]: 1.57–1.83), Females: a uric acid level of >6 mg/dl (OR: 2.35, 95% CI 1.83–2.99).

**Conclusion:**

Hyperuricemia was found to be an independent predictor of several lifestyle-related diseases, even after adjusting for WC which is closely related with insulin resistance. Hyperuricemia might require greater attention during the prevention of lifestyle-related diseases and future cardiovascular disease.

## Background

Hyperuricemia/gout tends to increase in all over the world [1–3]. In the United States, the person coming to the hospital for arthritis due to the gout tends to increase [4]. In recent years, in also Japan and Asian countries, hyperuricemia increases due to the westernization of food. Hyperuricemia is increased at all age in men 1996–2004 in Japan [5]. In addition, individuals with hyperuricemia often present with other lifestyle-related diseases, such as hypertension, hyperlipidemia, and diabetes [6–11]. Hyperuricemia is also expected to increase the incidence of ischemic heart disease and chronic kidney disease (CKD) [8, 12–15].

In recent several decades, metabolic syndrome (Mets) has been received considerable attentions [16]. Mets, which can be caused by increases in visceral fat accumulation, is also associated with the same lifestyle-related diseases due to insulin resistance [17]. Visceral fat tissue secretes tumor necrosis factor-α, plasminogen activator inhibitor-1, and free fatty acids, triggering blood vessel changes and insulin resistance [18]. In addition to classical components of Mets, a link between hyperuricemia and Mets has been reported [19, 20], and the exacerbation of insulin resistance due to increases in visceral fat accumulation can cause hyperuricemia by reducing renal uric acid secretion [21]. Studies have also shown hyperuricemia to be a predictor of future Mets [22, 23].

Conversely, another study found that treating hyperuricemia reduced the prevalence of lifestyle-related diseases among hyperuricemic rats [24]. Hyperuricemia might be related to lifestyle-related diseases and cardiovascular disease independently of the effects of increased visceral fat accumulation [12, 13].

Although hyperuricemia are closely linked to lifestyle-related diseases, the details of these links are complex and largely unknown. We therefore conducted a study to clarify one part of the actual relationship between hyperuricemia and lifestyle-related diseases after adjusting with waist circumference (WC) closely reflected with insulin resistance.

## Methods

### Participants

This was a cross-sectional study conducted at the Center for Preventive Medicine, NTT Kanto Medical Center, Tokyo, from May 2006 to March 2015. We run a comprehensive medical examination institution, which provides health check-up programs. Employers in Japan are required by The Industrial Safety and Health Law to commission medical examinations once a year to ensure the health of their employees. NTT, a telecommunications company, have entered into a contract in which we provide medical examinations based on the requirements of the abovementioned law to their employees.

Almost all of the study subjects were volunteers obtained from among the employees of NTT and their families. Therefore, most of the subjects were male and ranged in age from 40- to 60-years-old.

### Variables

This large-scale cross-sectional study was conducted as part of our general health check-up program. We provide several health check-up packages, which include various medical examinations, such as blood tests, electrocardiograms, gastrofiberscope, computed tomography scans, and other tests. Our program also includes many types of blood test that are not mandated by the abovementioned law. All examinations were performed by the same trained staff at a single institution. Some of the results for some subjects were used for the employee medical examinations mandated by The Industrial Safety and Health Law of Japan. Therefore, the precision of the examinations remained constant.

The subjects completed self-administered questionnaires about their demographic characteristics, medical history (diabetes, hypertension, dyslipidemia, and hyperuricemia/gout), and lifestyle habits (alcohol consumption, smoking status, and physical activity), and then well-trained staff interviewed any subjects who had failed to complete their forms. Weight and height were measured after the removal of shoes and heavy clothing. Also, WC was measured at umbilical level [25]. Blood pressure was measured in the sitting position with an automatic monitor after 15 min rest. Serum and urine samples were collected from each subject after overnight fasting and immediately subjected to biochemical analysis. Blood was drawn and used to determine the fasting cholesterol level, serum uric acid concentration, fasting plasma glucose (FPG) concentration, and serum creatinine concentration. In the case of subjects who underwent several examinations during the study period, the latest data were analyzed.

The subjects’ clinical data were retrospectively retrieved from an institutional database. Before each examination, all subjects were informed that the clinical data obtained by the program might be retrospectively analyzed and published. All of the examinations included in this study were performed as a routine part of the program, and none were aimed at specifically collecting data for the current study. The subjects’ records/information were anonymized and de-identified prior to the analysis. The study protocol was approved by the institutional ethics committee of NTT Kanto Medical Center.

The exclusion criteria were as follows: subjects with a lifestyle-related disease (hypertension, diabetes, dyslipidemia, hyperuricemia, or gout) and subjects for whom insufficient data were available.

In men, hyperuricemia was defined as a uric acid level of ≥7 mg/dl based on the Japanese Society of gout and nucleic acid metabolism criteria [5]. As female subjects with a uric acid level of >7 mg/dl were very few, we defined female subjects with a uric acid level >6 mg/dl as hyperuricemia according to previous announcement. [26] Mets components were defined using the following the Japanese metabolic syndrome criteria [25, 27]; high blood pressure: a systolic blood pressure level of ≥130 mmHg or a diastolic blood pressure level of ≥85 mmHg; impaired glucose tolerance: an FPG level of ≥110 mg/dl; dyslipidemia: a triglyceride level of ≥150 mg/dl or a high-density lipoprotein cholesterol level of <40 mg/dl. The examined lifestyle factors were defined as follows: alcohol consumption: drinking alcohol once or more a week, smoking: being a current smoker, and physical activity: exercising less than once a week.

### Statistical analysis

We analyzed the data for each sex separately. The subjects were divided into two categories according to the presence/absence of hyperuricemia. Their demographic characteristics were then compared between the groups with or without hyperuricemia using the Chi square test. Subsequently, the subjects were stratified into quartiles according to their WC as follows: males: Q1 (<78.4 cm), Q2 (78.4 to <83.5 cm), Q3 (83.5 to <89 cm), and Q4 (≥89 cm); females: Q1 (<71.6 cm), Q2 (71.6 to <77 cm), Q3 (7 to <83.2 cm), and Q4 (≥83.2 cm). The relationships between these quartiles and the presence of ≥2 components of metabolic syndrome or hyperuricemia were then evaluated using Chi square analysis.

The factors that were significantly associated with the presence of ≥2 components of Mets were then determined using multivariate logistic regression analysis. The covariates examined in the multivariate analysis were age, the presence of hyperuricemia, WC quartile, and lifestyle habits.

All calculations were performed using the JMP PRO software, version 11.0 (SAS Institute, Cary, NC, USA). Continuous data are reported as the mean ± SD. P values of <0.05 were considered to be statistically significant.

## Results

**Fig. 1:**
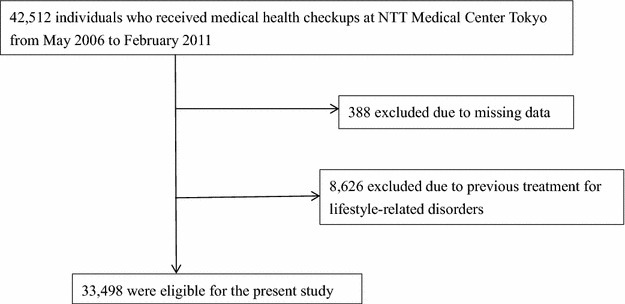
Participants’ registration flow

According to the exclusion criteria, 8626 subjects who were treated for lifestyle-related diseases and 388 whose data were insufficient were excluded. Thus, 33,498 subjects (males: 71.5%; mean age: 47 ± 11 years) were eligible for the study (inclusion rate: 78.8%) (Fig. 1).

Table 1 shows the characteristics of the study subjects. The mean age of the males was 47 years, and the mean age of the females was 48 years. The mean WC of the males was 84 cm, and their mean BMI was 23 kg/m^2^. The mean WC and BMI of the females were 78 cm and 21 kg/m^2^, respectively. A total of 2972 (12%) males and 559 (6%) females were considered to have impaired glucose tolerance; 9060 (38%) males and 2006 (21%) females were shown to have high blood pressure; 6031 (25%) males and 616 (6%) females exhibited dyslipidemia; 5869 (25%) males and 617 (7%) females were found to have hyperuricemia; and 4400 (18%) males and 536 (6%) females demonstrated multiple components of Mets.

**Table 1:** Demographic characteristics

Items	Mean (±standard deviation) or N (%)	
	Male (n = 23,945)	Female (n = 9553)
Age (years)	47 ± 11	48 ± 12
Body mass index (kg/m^2^)	23.3 ± 3.0	21.3 ± 3.2
Waist circumference (cm)	84.0 ± 8.3	78.1 ± 9.0
*Blood pressure measurements (mmHg)*		
Systolic	124 ± 16	114 ± 18
Diastolic	79 ± 10	72 ± 11
Subjects with high blood pressure	9060 (38)	2006 (21)
*Lipid metabolism*-*related measurements*		
High-density lipoprotein cholesterol (mg/dl)	56 ± 14	69 ± 15
Triglycerides (mg/dl)	119 ± 90	79 ± 45
Subjects with dyslipidemia	6031 (25)	616 (6)
Glucose metabolism-related measurements		
Fasting plasma glucose (mg/dl)	100 ± 15	95 ± 10
Subjects with impaired glucose tolerance	2972 (12)	559 (6)
*Uric acid metabolism*-*related measurements*		
Uric acid (mg/dl)	6.3 ± 1.2	4.6 ± 1.0
Subjects with hyperuricemia	5869 (25)	115 (1)
Subjects with ≥2 components of Mets	4400 (18)	536 (6)
*Lifestyle*-*related items*		
Alcohol intake (once or more a week)	15,442 (64)	3560 (37)
Current smoker	7332 (31)	892 (9)
Exercising (less than once a week)	15,033 (63)	6348 (66)

Mets, metabolic syndrome; the Mets components included high blood pressure, dyslipidemia, and impaired glucose tolerance

The subjects were categorized according to their uric acid levels and sex (Table 2). Both the male and female subjects with hyperuricemia displayed significantly higher prevalence rates of high blood pressure, dyslipidemia, impaired glucose tolerance, or ≥2 components of Mets than the subjects of the same sex with hyperuricemia.

**Table 2:** Percentage of subjects with various components of Mets stratified according to hyperuricemia

	Males		p value*	Females		p value*
	N (%)			N (%)		
	Uric acid >7 mg/dl	Uric acid ≤7 mg/dl		Uric acid >6 mg/dl	Uric acid ≤6 mg/dl	
Number of subjects	5869	18076		617	8936	
Subjects with high blood pressure	2735 (47)	6325 (35)	<0.01	238 (39)	1768 (20)	<0.01
Subjects with dyslipidemia	2299 (39)	3732 (21)	<0.01	134 (22)	482 (5)	<0.01
Subjects with impaired glucose tolerance	797 (14)	2175 (12)	<0.01	97 (16)	462 (5)	<0.01
Subjects with ≥2 components of Mets	1610 (27)	2790 (15)	<0.01	110 (18)	426 (5)	<0.01

Mets, metabolic syndrome; the Mets components included high blood pressure, dyslipidemia, and impaired glucose tolerance

* According to the Chi square test or Fisher’s exact test

As the subjects’ waist circumference increased, the prevalence rates of multiple components of Mets and hyperuricemia increased (Table 3). Furthermore, the prevalence of lifestyle-related diseases was significantly higher among the subjects with hyperuricemia regardless of their WC. In addition, hyperuricemia was a risk factor of multiple components of Mets significantly regardless of their WC (Table 4).

**Table 3:** Percentage of subjects with ≥2 components of Mets stratified according to waist circumference and hyperuricemia

Waist (cm)	<78.4		p value*	78.4 to <83.5		p value*	83.5 to <89.0		p value*	≥89.0		p value*
Uric acid levels (mg/dl)	>7	≤7		>7	≤7		>7	≤7		>7	≤7	
*Males*												
Number of subjects	800	5166		1212	4687		1602	4446		2255	3777	
Subjects with ≥2 components of Mets (%)	68 (9)	248 (5)	<0.01	187 (15)	503 (11)	<0.01	431 (27)	819 (18)	<0.01	924 (41)	1220 (32)	<0.01

Waist (cm)	<71.6		p value*	71.6 to <77.0		p value*	77.0 to <83.2		p value*	≥83.2		p value*
Uric acid levels (mg/dl)	>6	≤6		>6	≤6		>6	≤6		>6	≤6	
*Females*												
Number of subjects	64	2312		83	2277		132	2284		338	2063	
Subjects with ≥2 components of Mets (%)	0 (0)	17 (0.7)	1.00	6 (7)	48 (2)	<0.01	16 (12)	99 (4)	<0.01	88 (26)	262 (13)	<0.01

Mets, metabolic syndrome; the Mets components included high blood pressure, dyslipidemia, and impaired glucose tolerance

* According to the Chi square test or Fisher’s exact test

**Table 4:** Odds ratios of hyperuricemia for ≥2 components of Mets stratified according to waist circumference (univariate logistic regression analysis)

Waist (cm)	Odds ratio (95% confidence interval)							
	<78.4		78.4 to <83.5		83.5 to <89.0		≥89.0	
*Males*								
Uric acid >7 mg/dl	1.84	(1.38–2.42)	1.52	(1.26–1.82)	1.63	(1.42–1.86)	1.46	(1.31–1.62)

Waist (cm)	Odds ratio (95% confidence interval)							
	<71.6		71.6 to <77.0		77.0 to <83.2		≥83.2	
*Females*								
Uric acid >6 mg/dl	–	–	3.62	(1.36–8.10)	3.04	(1.68–5.19)	2.42	(1.83–3.18)

Mets, metabolic syndrome; the Mets components included high blood pressure, dyslipidemia, and impaired glucose tolerance

The factors that were significantly associated with the presence of ≥ 2 components of Mets are shown in Table 5. The following factors were associated with the presence of ≥2 components of Mets in the multivariate regression analysis model for males: a uric acid level of >7 mg/dl (OR: 1.70, 95% CI 1.57–1.83), WC Q2 (OR: 2.13, 95% CI 1.85–2.45), Q3 (OR: 3.90, 95% CI 3.42–4.45), and Q4 (OR: 8.22, 95% CI 7.25–9.36). The following factors were associated with the presence of ≥2 components of Mets in the multivariate regression analysis model for females: a uric acid level of >6 mg/dl (OR: 2.35, 95CI 1.83–2.99), WC Q2 (OR: 3.00, 95% CI 1.77–5.36), Q3 (OR: 5.20, 95% CI 3.20–9.02), and Q4 (OR: 14.16, 95% CI 8.90–24.14).

**Table 5:** Factors associated with the presence of ≥2 components of Mets (binary logistic regression analysis)

Variables	Odds ratio (95% confidence interval)							
	Males				Females			
	Univariate analysis		Multivariate analysis^a^		Univariate analysis		Multivariate analysis^a^	
Hyperuricemia	2.07	(1.93–2.22)	1.70	(1.57–1.83)	4.33	(3.44–5.43)	2.35	(1.83–2.99)
*Waist circumference quartiles for males (cm)*								
<78.4	Reference		Reference					
78.4 to <83.5	2.37	(2.06–2.72)	2.13	(1.85–2.45)	–	–	–	–
83.5 to <89.0	4.66	(4.10–5.31)	3.90	(3.42–4.45)	–	–	–	–
≥89.0	9.86	(8.71–11.19)	8.22	(7.25–9.36)	–	–	–	–
*Waist circumference quartiles for females (cm)*								
<71.6					Reference		Reference	
71.6 to <77.0	–	–	–	–	3.25	(1.92–5.79)	3.00	(1.77–5.36)
77.0 to <83.2	–	–	–	–	6.94	(4.27–11.99)	5.20	(3.20–9.02)
≥83.2	–	–	–	–	23.68	(14.98–40.19)	14.16	(8.90–24.14)
*Lifestyle-related items*								
Alcohol intake (once or more a week)	1.27	(1.19–1.37)	1.20	(1.11–1.29)	0.93	(0.77–1.11)	1.09	(0.90–1.33)
Current smoker	1.30	(1.21–1.39)	1.26	(1.17–1.36)	1.19	(0.89–1.56)	1.23	(0.90–1.66)
Exercising (less than once a week)	1.11	(1.04–1.19)	1.12	(1.04–1.21)	0.85	(0.71–1.02)	1.13	(0.93–1.38)

Mets, metabolic syndrome; the Mets components included high blood pressure, dyslipidemia, and impaired glucose tolerance

^a^ Adjusted for age

## Discussion

In the present study, the prevalence of hyperuricemia increased with WC in both sexes. After adjusting for WC, age, and lifestyle habits, hyperuricemia was revealed to be an independent predictor of several lifestyle-related diseases. To the best of our knowledge, this is the first study to clearly show direct relationships between hyperuricemia and lifestyle-related diseases which are components of Mets, even after accounting for the effects of increased WC which reflects insulin resistance.

Previous studies have shown that hyperuricemia is linked with Mets [19, 20, 28]. It is possible that the exacerbation of insulin resistance brought about by the increases in visceral fat accumulation that underlie Mets causes hyperuricemia by reducing renal uric acid secretion [21]. In the present study, the prevalence of hyperuricemia increased with WC in both sexes suggesting that a relationship exists between hyperuricemia and increased visceral fat accumulation. Moreover, several previous studies have detected relationships between hyperuricemia and lifestyle-related diseases [6–11]. This finding indicates that hyperuricemia might exacerbate lifestyle-related diseases independently of visceral fat accumulation which reflects insulin resistance.

Relating with above discussion, several previous reports indicated the association between hyperuricemia and lifestyle-related disorders. A large scale cross sectional study, which analyzed the 85,000 Japanese workers, showed that the person with hyperuricemia were 1.79 times likely to have hypertension in men than normal level of uric acid, and almost 6 times in the women [9]. Kodama et al. reported that 11 of cohort study showed that serum uric acid level was associated with development of type 2 diabetes mellitus [21]. As for the underlying mechanism responsible for these effects, it has been suggested that hyperuricemia reduces nitric oxide production in the vascular endothelium and increases the concentrations of renin and angiotensin, thereby impairing renal blood flow and triggering high blood pressure [29]. Thus, it may be repaired to pay attention to hyperuricemia for prevention of lifestyle related disorders.

Sex hormones play a role in these differences, and females (particularly premenopausal females) often have lower uric acid levels than males [30]. In our study, although there were not many female subjects with hyperuricemia or multiple lifestyle-related diseases, those with hyperuricemia appeared to be at higher risk of comorbidities, including several lifestyle-related diseases, irrespective of their visceral fat accumulation. A Korean report indicated that subjects who had high uric acid level people were likely to become Mets, and the impact in females may be than men [31].

Thus, females with hyperuricemia might require special attention as well as males.

## Limitations

Our study has several limitations. First, this study is a cross-sectional observational study based on data from a single institution, and the results are therefore limited in their applicability to all hyperuricemia patients in Japan. In addition, important measurements, such as immuno-reactive insulin (IRI), to estimate Homeostasis model assessment-Insulin Resistance (HOMA-IR) were not collected. Causal relationship of hyperuricemia, WC, and/or life-style related diseases remain unclear. Further multicenter collaborative research is needed. Second, our study was affected by selection bias. Approximately 70% of our participants were healthy male office workers who ranged in age from 40 to 60-years-old. In addition, there were a few observed female participants in our study, and our study does not consider the influence of the menopause. Thus, this limited sample might not have accurately represented the whole population. Lastly, lifestyle habits were evaluated using a self-administered questionnaire and so the participants might have stated that they had more healthy lifestyles than they actually did. Thus, it is possible that the participants were not as healthy as our data suggested.

## Conclusion

Hyperuricemia was shown to be linked to increased abdominal obesity and other lifestyle-related conditions and to make an important contribution to Mets. In addition, hyperuricemia was found to be an independent predictor of multiple lifestyle-related diseases, even after accounting for increased abdominal obesity which reflected with insulin resistance. Hyperuricemia might require greater attention during the prevention of lifestyle-related diseases and future cardiovascular disease.
